# Making Nursing Visible in General Practice: Responsible Action Research as an Approach to Developing Nurse‐Sensitive Metrics

**DOI:** 10.1111/nin.70130

**Published:** 2026-06-28

**Authors:** Orla Loftus Moran, Mary Casey

**Affiliations:** ^1^ School of Nursing, Midwifery and Health Systems University College Dublin Dublin Ireland

**Keywords:** Action Research, Appreciative Inquiry, general practice nursing, nurse‐sensitive indicators, nursing metrics, primary care, Responsible Action Research

## Abstract

This critical discussion paper argues that the development of nurse‐sensitive metrics in general practice is not simply a technical task of selecting indicators, but an ethical and methodological process through which nursing contribution becomes known, named and represented. Although health systems increasingly prioritise measurement, accountability and performance data, important dimensions of nursing practice remain poorly captured, particularly in community and primary care contexts. In general practice, nursing care is characterised by generalist, relational, preventive and cumulative forms of care, making the direct transfer of metrics developed outside this context problematic. General practice nurses represent a substantial workforce, yet their contribution remains only partially visible within existing healthcare data systems. This paper advances a Responsible Action Research approach to developing nurse‐sensitive metrics grounded in everyday practice. Drawing on Action Research, implemented through Appreciative Inquiry and informed by the Quality Action Research Checklist, the paper considers how collaborative inquiry can support general practice nurses to articulate dimensions of nursing quality that may otherwise remain tacit. Inclusion of perspectives from general practitioners, patients and practice administrators further situates this inquiry within the broader ecology of care. The paper argues that responsible metric development requires attention not only to what is eventually measured, but to the transparency, reflexivity and accountability of the process through which potential indicators are generated. By reframing metric development as a practice‐engaged process of knowledge generation, it offers a methodological contribution to debates on how nursing work can be made visible without reducing its complexity.

## 1 Introduction: Lack of Visibility of Nursing

To date, no research has been identified in Ireland examining the development of nurse‐sensitive metrics specific to general practice nursing (Loftus Moran and Casey 2025). International research has similarly highlighted challenges in developing nurse‐sensitive metrics capable of representing nursing practice within community and primary care settings (J. Lukewich et al. 2018). This gap reflects the wider invisibility of general practice nursing within healthcare quality and performance systems. Health systems internationally are increasingly relying on primary care to respond to the complex health needs associated with ageing populations, rising chronic disease prevalence and escalating healthcare costs. Primary healthcare is also positioned as central to progress toward universal health coverage and the Sustainable Development Goals (World Health Organization and United Nations Children's Fund 2018; World Health Organization 2008, 2018; Coy and Tanwir 2025). Well‐developed primary care systems are associated with improved population health outcomes, more responsive services and greater cost effectiveness. Within these systems, general practice represents a key organisational setting through which primary care is delivered, providing accessible, continuous and coordinated care across the life course (Starfield et al. 2005; D. Kringos et al. 2013; Akman et al. 2022). Registered nurses are increasingly embedded within primary care teams and contribute across a broad spectrum of activities, including chronic disease management, preventive and screening services, patient education and self‐management support, and coordination of care within multidisciplinary primary care teams (Beattie et al. 2026; Freund et al. 2015; Norful et al. 2017; International Council of Nurses 2024). Despite this, the contribution of general practice nurses (GPNs) remains only partially visible within healthcare performance and data systems (J. Lukewich et al. 2021).

The limited visibility of nursing work is not simply a technical gap in measurement but reflects a deeper challenge concerning how knowledge about practice is generated, represented and valued. Across health systems internationally, measurement has become central to how healthcare quality is evaluated and improved, with indicators and performance metrics increasingly shaping policy decisions, resource allocation and professional accountability (D. S. Kringos et al. 2010; World Health Organization and the United Nations Children's Fund 2022). This raises questions about how clinical relationships, professional judgement and continuity of care can be represented within performance measurement frameworks (Greenhalgh and Papoutsi 2018). This challenge is particularly evident in general practice, where indicators have expanded considerably in recent years (Chambers et al. 2025). Such approaches tend to prioritise what can be readily counted, often overlooking the relational, interpretive and context‐dependent dimensions of care that are central to general practice nursing. This creates a risk that what matters most in sustaining patient care such as continuity, trust, professional judgement and relational engagement remains underrepresented. To understand why the development of nurse‐sensitive metrics within general practice remains challenging, it is necessary to examine how nursing quality has been conceptualised and measured within the wider healthcare literature.

While the argument developed in this paper has relevance beyond a single health system, our interest arose from the Irish general practice context. In Ireland, general practice is a key setting for primary care, with general practitioners acting as the first point of contact and as gatekeepers to a wide range of health and social services, while working within practice teams that include GPNs and midwives and practice administrators (Coy and Tanwir 2025). General practice also occupies a distinctive position within the Irish health system as a largely privately organised sector in which the State contracts for some services (Coy and Tanwir 2025). As a result, national data on general practice workforce, activity and workload are fragmented, contributing to an opaque data landscape. This data opacity is directly relevant to the argument developed in this paper, as it highlights the difficulty of representing nursing contribution within existing healthcare data and performance systems. Given this Irish general practice context, we use the term primary care to refer to the community‐based service setting in which general practice care is delivered, while recognising that primary healthcare is used more broadly in international literature to describe a whole‐society approach encompassing service provision, public health, multisectoral action and population empowerment (Chotchoungchatchai et al. 2020).

This paper enhances scholarly publishing in nursing by critically exploring how Responsible Action Research supports the development of nurse‐sensitive metrics in general practice. In doing so, it argues that the development of meaningful metrics requires attention not only to outcomes, but to the ethical, relational and reflexive processes through which knowledge about nursing practice is generated, interpreted and made visible.

## 2 Quality Indicators and Nursing Metrics

Within healthcare quality measurement, indicators are generally understood as clearly defined aspects of care relating to structures, processes or outcomes (Campbell et al. 2003; Donabedian 2005). Indicators therefore describe the dimension of care being assessed, while metrics refer to the specific measures through which those indicators are operationalised and quantified in practice (Foulkes 2011). Within international literature, nurse‐sensitive indicators have emerged as measures intended to capture aspects of care that are directly influenced by nursing practice (Afaneh et al. 2021; Heslop et al. 2014; Oner et al. 2021; Teeling et al. 2021). Much of the development of nurse‐sensitive indicators has taken place within acute care environments, where measurement frameworks align more readily with discrete clinical processes, procedural and safety outcomes and standardised care activities (Gormley et al. 2024; Heslop et al. 2014; Oner et al. 2021). The development of such indicators is typically informed by conceptual frameworks and structured indicator development processes designed to ensure that performance measures are clearly specified, valid and comparable across healthcare settings (Donabedian 1988; Dubois et al. 2013; Riehle et al. 2007).

While indicators and metrics play an important role in supporting accountability and quality improvement, they also shape how healthcare quality is defined, represented and understood within healthcare systems (Porter 2010). The rapid expansion of quality indicators has raised concerns that extensive measurement may burden clinicians while offering limited insight into improving care (Marang‐van de Mheen and Vincent 2023). This concern is particularly relevant in primary care settings, where aspects of care central to sustaining patient well‐being, including continuity, relational engagement and clinical judgement developed over time, may be difficult to capture through discrete, standardised indicators (Arvidsson et al. 2019). Much of this work is interpretive, relational and cumulative, emerging across encounters and shaped by contextual knowledge of patients and practice (Chan et al. 2024; Prior et al. 2025; Stange et al. 2014). Measurement systems tend to prioritise what can be readily counted rather than what may be most central to sustaining care (Greenhalgh and Papoutsi 2018). As a result, important dimensions of care may remain only partially represented within routine measurement systems, reflecting the recognised challenges of developing quality indicators that adequately capture the complexity of general practice care (Arvidsson et al. 2019; ni Riain et al. 2015).

Efforts to measure the quality and impact of nursing care have developed over several decades as health systems have sought to better understand the contribution of nursing to patient outcomes and service performance (Montalvo 2007). However, in primary and community care settings, the development of nurse‐sensitive metrics remains fragmented and conceptually inconsistent (Morgan‐Gorman et al. 2024; Rapin et al. 2015; L. A. Siaki et al. 2023). Consequently, important dimensions of nursing care in general practice remain poorly represented in measurement systems. General practice performance is inherently multidimensional, encompassing clinical care, access, continuity, patient experience and organisational functioning, making the development of representative indicators particularly complex (Chambers et al. 2025). Within this clinical setting, GPNs contribute across a broad and generalist spectrum of care. This includes the management of chronic illness, the delivery of preventive and screening programmes, therapeutic monitoring, patient education and self‐management support, and the coordination of care within multidisciplinary primary care teams (Beattie et al. 2026; Bury et al. 2021; Casey, O'Connor, et al. 2022; Clifford et al. 2021). This generalist and longitudinal orientation of care distinguishes general practice nursing from hospital‐based models of care and complicates the direct translation of nurse‐sensitive indicators developed in acute care settings into general practice contexts (Mastal et al. 2016; Morgan‐Gorman et al. 2024).

In Ireland, the need to make nursing contribution more visible sits within a wider policy emphasis on performance measurement, digital health infrastructure and workforce intelligence (Department of Health 2017; Health Service Executive 2024; O'Connor et al. 2020). The Health System Performance Assessment (HSPA) framework (D. Kringos et al. 2021) further positions performance measurement as central to Sláintecare health policy reform (Burke et al. 2018). National health policy also highlights the importance of strengthening data systems capable of supporting this monitoring. The National Digital Health Strategy (Department of Health 2024a) emphasises the development of integrated digital health records and data infrastructure to support healthcare delivery, workforce capability and system performance measurement. Policy documents in the context of nursing regulation similarly highlight the need to strengthen workforce intelligence and data infrastructure to better understand nursing activity and contribution within health services. The Report of the Expert Review Body on Nursing and Midwifery (Department of Health 2022) identified significant limitations in available data describing nursing activity and recommended the development of more coherent national data systems to support workforce planning and service evaluation. More recently, the Strategy for the Office of the Chief Nursing Officer 2024–2026 (Department of Health 2024b) reiterates the importance of strengthening data systems to support clearer understanding of nursing activity and impact. Internationally, the World Health Organization (2025) State of the World's Nursing report similarly reflects a growing expectation that nursing contribution be systematically documented to inform workforce planning and health system performance.

The general practice nursing workforce represents a substantial component of primary care service delivery in Ireland, with an estimated 2288 nurses providing approximately 5.7 million consultations annually across general practice settings (S. Connolly and Flanagan 2024; S. Connolly et al. 2025). However, despite the scale of this contribution, the work of nurses in general practice remains only partially visible within routine data systems. National workforce analysis highlights significant limitations in available data describing the scale, scope and activities of nurses working in general practice in Ireland (S. Connolly and Flanagan 2024). Researchers have increasingly explored how nursing care might be conceptualised and measured within primary and ambulatory care settings (Halcomb et al. 2017; Langins and Borgermans 2015; J. Lukewich et al. 2018; J. A. Lukewich et al. 2019). Within this broader literature, indicators relating to nursing care in primary and ambulatory care settings have been proposed across areas such as continuity, care coordination, patient education and chronic disease management (American Academy of Ambulatory Care Nursing 2016; Rapin et al. 2015; L. Siaki et al. 2022). However, indicators and metrics remain inconsistently defined and variably operationalised in general practice contexts, reflecting the ongoing challenge of developing measurement approaches capable of capturing the reality of nursing care in these settings (Loftus Moran and Casey 2025; Morgan‐Gorman et al. 2024).

## 3 Methodological Approaches and Frameworks for Developing Nursing Metrics

A range of methodological approaches have been used to develop healthcare quality indicators, including Delphi consensus studies (Boulkedid et al. 2011), expert panel development (Brook et al. 2000), framework‐based indicator construction (Donabedian 2005) and mixed‐methods approaches involving clinicians and stakeholders (Campbell et al. 2003). Similar approaches have been applied within nursing research to develop nurse‐sensitive indicators and nursing quality metrics (D. Connolly and Wright 2017; Dubois et al. 2013; Koch et al. 2020; Maben et al. 2012; Marmo et al. 2021; O'Connor et al. 2022; Sim et al. 2018). Within primary and ambulatory care contexts, indicator development studies have also drawn on consensus methods and expert‐informed frameworks to identify potential measures of nursing contribution (Dufour et al. 2018; J. Lukewich et al. 2020; Mathews et al. 2021; Morgan‐Gorman et al. 2024; Rapin et al. 2015; L. Siaki et al. 2022; Strasser et al. 2005). While these approaches have generated important insights, they often rely on expert judgement or secondary analysis of practice rather than direct engagement with clinicians examining everyday care within its organisational context. For general practice nursing, the absence of contextually relevant, nurse‐sensitive metrics represents both a professional and methodological challenge. Previous efforts to develop quality indicators for Irish general practice have largely focused on organisational and practice‐level processes, reflecting the complexity of measuring quality within a generalist primary care setting (ni Riain et al. 2015). However, these approaches do not specifically address how the contribution of nurses within general practice teams might be articulated or measured.

This gap is not simply technical. Researchers have argued that the generalist, longitudinal and relational nature of primary care requires measurement approaches capable of capturing dimensions of care that extend beyond discrete clinical activities (Stange et al. 2014). Studies exploring patient perspectives on general practice reinforce this point, highlighting the importance of therapeutic relationships, trust, familiarity and the capacity of clinicians to listen attentively (Brennan et al. 2019). Within nursing, related concerns have been raised regarding the difficulty of making nursing practice visible through conventional measurement systems, particularly in ambulatory and community care contexts (American Academy of Ambulatory Care Nursing 2016; Morgan‐Gorman et al. 2024; Rapin et al. 2015).

According to Teeling et al. (2021), the usefulness of nursing metrics depends on their alignment with everyday clinical practice and the active engagement of nurses in their development and implementation. In community‐based primary care settings, nursing practice develops through ongoing relationships with patients and through the coordination, education and preventive work that supports the functioning of the practice (J. Lukewich et al. 2020). Understanding what constitutes quality nursing contribution in this context must precede attempts to measure it. This requires approaches that engage those who are closely connected to practice in processes of reflection and inquiry, recognising that knowledge about practice develops through dialogue and collaborative examination of experience (Bradbury‐Huang 2010; Coghlan 2019a). Action Research offers a methodological framework capable of supporting such practice‐engaged inquiry. It provides a structured approach to exploring how practice‐grounded indicators of nursing contribution may emerge from collaborative examination of everyday practice. Approaches such as Insider Action Research offer a distinct advantage in this regard, as it enables the process whereby the action research is conducted by a ‘full member’ of an organisational system, rather than by one who enters the system as an external researcher and remains only for the duration of the research (Coghlan 2019b). Hence, it facilitates practice nurses to examine and interpret everyday practice from within the settings in which care is delivered and where the practical knowledge of nursing practice is held.

Conceptual frameworks are commonly used to structure the development and interpretation of healthcare quality indicators (Senn et al. 2021). Donabedian (1988) widely cited model conceptualises quality in terms of structure, process and outcomes, providing a foundational logic for understanding how healthcare activities lead to measurable results. Subsequent work in nursing and health services research has further emphasised the importance of clearly articulated domains when seeking to capture the contribution of nursing practice to patient care and health system performance (Dubois et al. 2013). National guidance on performance measurement similarly highlights the importance of structured frameworks when developing indicators for nursing and midwifery services (Health Information and Quality Authority 2013). Work examining the development of nursing metrics emphasises that indicators must reflect aspects of care that are valued by clinicians and patients and that they must be credible and feasible within practice contexts (Maben et al. 2012). Addressing this challenge requires more than the development of new indicators; it requires reconsideration of how knowledge about nursing practice is produced.

Action Research offers a methodological orientation aligned with the central argument of this paper: that nurse‐sensitive metrics must emerge from inquiry into practice rather than from externally imposed frameworks. In this paper, Insider Action Research is proposed, not solely as a methodological choice but as a responsible approach to inquiry. It is particularly suited to complex healthcare contexts in which professional practice, organisational dynamics and service user experience are interwoven (Bradbury 2015; Greenwood 2015). Furthermore, Coghlan (2019b) conceptualises Action Research across three interrelated levels:

- First‐person inquiry, which involves the researcher's ongoing reflexive examination of his or her own assumptions and actions.
- Second‐person inquiry, which centres on collaborative dialogue and inquiry among participants addressing a shared issue.
- Third‐person inquiry, through which insights generated within the research setting are communicated to wider audiences.

This multi‐level structure enables knowledge to emerge through both collaborative engagement within the research process and the dissemination of insights beyond it. Action Research also recognises the knowledge embedded within professional practice, enabling practitioners to examine and articulate insights that may otherwise remain tacit within everyday work (Coghlan 2019b). It offers a framework through which knowledge about nursing practice is generated collaboratively, while remaining accountable to the relationships, contexts and processes through which that knowledge emerges. These principles are not simply procedural considerations; they shape how knowledge about practice is generated and, in turn, what can be effectively measured. In this sense, the development of nurse‐sensitive metrics is not treated as a technical exercise, but as a process of inquiry into what constitutes meaningful nursing contribution within general practice. However, the process must be rigorous. Rigour in Action Research is not secured through standardisation alone, but through explicit attention to context, relational engagement, process transparency and theory development (Casey et al. 2025). Attention to these dimensions positions Action Research not merely as a method for developing metrics, but as a means of interrogating how nursing contribution becomes knowable and measurable within general practice. Its dual orientation toward practical improvement and conceptual development is therefore critical, enabling the generation of metrics that are both contextually grounded and theoretically informed. In doing so, the approach directly addresses the limitations of existing indicator development strategies, which often abstract practice from the settings in which it is constituted. Therefore, it is not just about using Action Research; it is about making explicit its responsibility dimension. Responsible Action Research provides a framework for this reconsideration by foregrounding the ethical and relational foundations of inquiry as integral to methodological practice.

## 4 Responsible Action Research

Responsible Action Research draws on traditions of participatory, reflexive and quality‐oriented Action Research (Coghlan 2019b; Reason and Bradbury 2008). Within this perspective, research is accountable not only for the outcomes it generates but also for the processes through which knowledge is co‐created. This shifts attention from the production of metrics as technical artefacts toward the conditions under which meaningful and credible knowledge about practice can emerge. Central to this approach is the inclusion of interest‐holders as active participants in the research process. In the context of general practice, this involves engaging nurses, general practitioners, patients and practice staff in collaborative inquiry into everyday care. Through such engagement, knowledge is generated dialogically, enabling multiple perspectives to shape how nursing contribution is understood and articulated. This participatory orientation also supports anticipation, where current practices are examined not only for what they achieve but for how they shape future possibilities for care, measurement and service development. Responsible Action Research further emphasises responsiveness and reflexivity within the research process. As inquiry unfolds, participants are encouraged to question taken‐for‐granted assumptions about practice, measurement and value, while remaining open to emerging insights and changing circumstances. This iterative and adaptive process recognises that understanding develops over time and that meaningful indicators cannot be predefined but must emerge through sustained engagement with practice. At the same time, attention to sustainability ensures that the development of metrics does not privilege short‐term visibility over longer term integrity of care, avoiding approaches that may distort practice or undermine future service provision.

Responsible Action Research prioritises a process of care within inquiry itself (Møller 2025). The relationships through which research is conducted are not neutral but constitute the conditions through which knowledge is formed. Creating spaces characterised by trust, openness and mutual respect enables participants to examine practice together and to articulate aspects of care that may otherwise remain implicit. In this sense, the research process becomes not only a means of generating knowledge but also a site in which ethical and relational commitments are enacted and sustained. Situated within this framework, this paper examines how a practice‐engaged, Responsible Action Research approach can support the development of nurse‐sensitive metrics in general practice. Rather than beginning with predefined indicators, it argues that meaningful metrics must emerge from collaborative inquiry into everyday practice, so that what is measured reflects the realities, values and complexities of nursing care.

### 4.1 Responsible Action Research in the Development of Nursing Metrics in General Practice

Given the generalist and context‐dependent nature of general practice nursing, approaches that design indicators from the top‐down risk overlooking important aspects of routine clinical work (Morgan‐Gorman et al. 2024; Rapin et al. 2015). Healthcare delivery is increasingly recognised as a complex adaptive system in which outcomes emerge through interactions between professionals, patients and organisational contexts (Greenhalgh and Papoutsi 2018). Consequently, research approaches that engage those directly involved in care delivery are increasingly advocated as a means of generating knowledge that remains closely connected to practice (Bradbury‐Huang 2010; Ciasullo et al. 2026). In the context of general practice, understanding practice requires approaches that engage directly with those involved in delivering and experiencing care.

Responsible Action Research highlights the ethical and relational foundations of research practice and offers a participatory and iterative framework through which action and knowledge generation occur concurrently, integrating reflection, action and theory development through engagement with practice (Coghlan and Brydon‐Miller 2014). In healthcare contexts, its growing application reflects its capacity to link theory and practice while engaging practitioners directly in generating actionable knowledge through collaborative inquiry (Casey, Coghlan, et al. 2022; Casey et al. 2021). By involving GPNs as collaborative participants in cycles of reflection, dialogue and design, while incorporating perspectives from general practitioners, patients and practice administrators, Action Research enables shared understandings of practice to be articulated and refined. Through this process, dimensions of nursing quality that may otherwise remain implicit within everyday practice can be articulated and explored as the basis for developing nurse‐sensitive metrics. In this way, it emphasises that inquiry is accountable not only for the outcomes it produces but also for how those outcomes are generated. This includes attention to who participates in the inquiry, how different perspectives are engaged, how assumptions are questioned and how emerging understandings may shape future practice.

### 4.2 Contextual, Relational and Participatory Foundations of the Action Research Inquiry

The Quality Action Research Checklist (QuARC) has been proposed as a framework for making rigour in Action Research explicit through attention to context, relational engagement, process transparency and theory development (Casey et al. 2025). These four dimensions are mobilised to reframe how nursing metrics in general practice might be developed. First, *context* is foregrounded by situating the inquiry within general practice settings where nursing care is enacted, recognising that quality is inseparable from the conditions in which care is delivered. Second, *relational engagement* extends the inquiry beyond nurses to include general practitioners, patients and practice administrators. These are not positioned as subjects of study but as contributors to a shared process of inquiry. Through this engagement, knowledge about nursing practice is generated dialogically, reflecting the interdependent nature of care within general practice teams. This participatory orientation enables multiple forms of knowing to be brought into conversation thereby ensuring that emerging understandings of nursing contribution are grounded in the realities of practice. Hence, the third dimension of *process transparency* is enacted through iterative cycles of dialogue and reflection, making visible how understandings of nursing practice are negotiated and refined over time. At the same time, participation is understood as an ethical commitment rather than a procedural step. Creating conditions for meaningful engagement requires attention to trust, openness and mutual respect, recognising that the quality of relationships shapes both the process and outcomes of the research. In this way, the inquiry itself becomes a site in which the relational foundations of care are enacted. Finally, *theory development* is positioned not as an abstract outcome but as an emergent process through which dimensions of nursing quality are articulated and translated into potential nurse‐sensitive indicators.

### 4.3 Researcher Positioning as an Insider and Reflexive Responsibility

In Insider Action Research, the practitioner–researcher position is central to how inquiry is shaped, particularly when the researcher is located within the field of practice being examined. Such positioning offers important methodological advantages, including contextual understanding of organisational routines, professional relationships and everyday practice (Coghlan 2019b). At the same time, it also introduces responsibilities concerning how knowledge is generated and interpreted. Insider knowledge cannot be treated as neutral access to practice; rather, it requires disciplined reflexive attention to how understanding is formed from within the setting. Drawing on Coghlan's (2024b) concept of the ‘data of consciousness’, reflexivity extends beyond reflection on observable events to include examination of how the researcher experiences, understands, judges and acts within the inquiry process. This form of reflexive engagement is central to Responsible Action Research because it links practical relevance with methodological rigour, supporting the emergence of knowledge from practice. Because Insider Action researchers work from within familiar professional contexts and existing relationships, the processes through which interpretations are formed and refined must be made transparent (Coghlan 2019b).

The Insider Action Researcher position therefore heightens the importance of relational and reflexive responsibility. Coghlan (2024a) suggests that the quality of collaborative relationships constitutes a ‘sweet spot’ in Insider Action Research, as inquiry depends on forms of engagement marked by openness, trust and shared exploration. In the development of nurse‐sensitive metrics, this matters because what becomes visible and measurable is shaped by the relationships, assumptions and interpretive processes through which practice is examined. Insider positioning does not seek to eliminate subjectivity; rather, it requires making visible how interpretation develops through the interaction between researcher, participants and context. In this sense, reflexive responsibility is not an additional safeguard but a core condition for generating credible, rigorous and practice‐grounded knowledge.

## 5 Structuring the Insider Action Research Inquiry: Appreciative Inquiry as a Responsible Process

While adopting an insider action research approach acknowledges the position of the action researcher to gain insider understanding and awareness of practice, the proposed structure of the action research approach is appreciative inquiry. Appreciative Inquiry provides more than a structure for participation within Responsible Action Research. In the development of nurse‐sensitive metrics, it offers a way of generating knowledge about practice that is affirmative, relational and interpretive. Rather than beginning with deficits, gaps or externally defined measures, Appreciative Inquiry directs attention to what practitioners recognise as meaningful, effective and valuable within everyday care. This is methodologically important because the purpose is not simply to identify measurable activities, but to clarify the dimensions of nursing quality that those activities may represent. Grounded in strengths‐based and constructionist traditions, Appreciative Inquiry focuses on identifying and building on what works well in practice (Bushe 2012; Cooperrider and Srivastva 2013). Within Insider Action Research, this orientation is closely aligned with humble inquiry, where engagement is guided by curiosity, openness and a willingness to explore practice with others rather than assume that its meaning is already understood (Coghlan 2024a). This stance is particularly relevant to the development of nurse‐sensitive metrics because it resists premature assumptions about what counts as nursing contribution and supports a more careful articulation of practice knowledge before it is translated into indicators and metrics.

The strengths‐based orientation of appreciative inquiry is organised through the 5D phases (Define, Discover, Dream, Design and Destiny), which guide participants through a process of collective reflection, envisioning and co‐creation (Watkins et al. 2011). Within this approach, these phases structure the collaborative process through which participants identify valued aspects of general practice nursing, envision what high‐quality nursing care might look like and co‐develop indicators capable of capturing these contributions within general practice settings.

The **Define** phase establishes the focus of inquiry and clarifies why making nursing contribution visible matters within the general practice context. The **Discover** phase supports examination of examples of effective nursing practice, enabling the clinical, relational, person‐centred and organisational dimensions of care to be identified. The **Dream** phase opens a space for imagining how these valued aspects of nursing might be more fully recognised, strengthened and represented. The **Design** phase supports translation of emerging understandings into potential domains and candidate indicators of nursing quality. The **Destiny** phase keeps attention on how such indicators might remain meaningful, feasible and accountable to the practice contexts from which they emerge.

Movement through these 5D phases is not linear but iterative. The process remains responsive to emerging insights, allowing understandings to be revisited and refined over time. This responsiveness reflects a key dimension of Responsible Action Research practice, ensuring that inquiry remains open to change rather than constrained by predetermined outcomes. Through successive cycles of reflection and dialogue, Appreciative Inquiry provides a forum in which everyday nursing roles can be interpreted collectively and translated into candidate domains of nursing quality. These domains may then inform the development of potential nurse‐sensitive indicators, understood not as fixed measures imposed on practice, but as developing representations of valued nursing contribution. Incorporating perspectives from general practitioners, patients and practice administrators can further broaden this process by situating nursing contribution within the wider ecology of general practice care. These perspectives help challenge a nursing‐only interpretation of contribution and support a more relational and organisational understanding of how nursing care is enacted, experienced and valued. In this way, Appreciative Inquiry supports responsible metric development by making visible not only what might be measured, but how potential indicators are generated, discussed, challenged and refined.

## 6 Developing and Interpreting Insights Within an Appreciative Inquiry Approach

In undertaking Responsible Action Research, knowledge is not extracted from practice but develops through cycles of reflection, dialogue and action within the inquiry process (Bradbury 2015; Coghlan and Brannick 2014). Through engagement with practice, Action Research generates what Coghlan (2011) describes as practical knowing, knowledge that emerges through collaborative inquiry and is tested in relation to practice. In the development of nurse‐sensitive metrics, this means that insight does not arise from externally categorising nursing activity, but from examining how nursing contribution is understood, enacted and valued within the setting where care is delivered.

Within this appreciative inquiry approach, like all other types of action research, documentation of dialogue, reflexive notes and interpretive decisions supports transparency in how understandings develop over time. These materials can be revisited across successive inquiry cycles, where interpretations are collectively examined and refined. Additional perspectives from general practitioners, patients and practice administrators can broaden this interpretive process by situating nursing care within the wider relational and organisational context of general practice. Through this collaborative interpretation, shared understandings of practice can be grouped into various categories describing key dimensions of nursing quality within general practice. These categories can provide a conceptual foundation from which nurse‐sensitive metrics can be responsibly developed.

Nursing metrics or indicators are therefore understood as emerging through collaborative reflection. In this way, metrics emerge not as predefined measures, but as representations of practice that have been collectively examined, negotiated and understood. Established quality frameworks can provide a useful interpretive lens for considering how emerging domains relate to broader understandings of healthcare quality. For example, Donabedian (1988) structure–process–outcome model offers a way of considering how emerging categories of nursing quality within general practice may be articulated as nurse‐sensitive indicators and subsequently translated into measurable metrics.

## 7 Demonstrating Rigour as Responsible Action Research Practice

Responsible Action Research requires that inquiry is accountable not only for the outcomes it generates but also for the processes through which knowledge is produced and applied. Questions concerning how rigour is demonstrated in Action Research have long been discussed, particularly in relation to transparency, systematicity and the articulation of how knowledge and change emerge through inquiry (Bradbury‐Huang 2010; Eden and Huxham 1996). Drawing on the QuARC, this paper uses the framework to consider how rigour can be made explicit within Responsible Action Research (Casey et al. 2025). In this paper, QuARC is not treated as an external checklist applied retrospectively, but as a way of organising responsibility across its four interconnected factors: context, relationships, inquiry process and outcomes. When applied to the development of nurse‐sensitive metrics, the QuARC highlights the need to consider not only whether indicators are meaningful and feasible but also how they are generated, whose knowledge informs them and what consequences they may have for care delivery, professional roles and the representation of nursing work within healthcare systems.

### 7.1 Responsibility for Context: Grounding Appreciative Inquiry in General Practice

Within the QuARC framework, attention to context involves examining the practical concern motivating the inquiry, the policy and professional environment in which it is situated and the extent to which existing research informs the inquiry. Action is driven by a practical concern arising from everyday experience within Irish general practice: while nurses contribute across a wide range of clinical, preventive and organisational activities (Bury et al. 2021), existing data systems offer limited mechanisms for representing this work within health system performance data (S. Connolly and Flanagan 2024). This concern is situated within the broader Sláintecare policy and service context in which health systems are increasingly seeking to strengthen primary care and shift services closer to the community (Kuhlmann et al. 2018; Oireachtas Committee on the Future of Healthcare 2017). Within this evolving policy landscape, understanding how the work of different professional groups is reflected in health system data has become increasingly important.

Existing research concerning nurse‐sensitive indicators, primary care performance measurement and the evolving role of nursing within general practice is an important contextual foundation for inquiry. A further contextual consideration involves engaging those directly involved in delivering and organising care within general practice, so that everyday nursing practice can be examined to see how it is enacted, interpreted and potentially represented through indicators and metrics grounded in practice experience. Together, these contextual elements demonstrate that context is not a background feature: it shapes the meaning, relevance and potential consequences of any metrics developed. The inquiry is therefore shaped by wider policy and professional developments; it builds on experiences and existing research relating to nurse‐sensitive indicators and primary care performance measurement.

### 7.2 Responsibility for Quality of Relationships in the Appreciative Inquiry: Participation, Collaboration and Reflexivity

A second consideration of rigour concerns the quality of relationships through which the inquiry develops. Within Responsible Action Research, relationships are not simply a means of data collection but constitute the conditions through which knowledge is generated. This requires attention to who participates, how they are engaged throughout the research process and whether participants act as co‐researchers in shaping emerging insights. Collaborative engagement is central to Action Research and shapes how understanding develops within the inquiry (Coghlan and Brannick 2014; Reason and Bradbury 2008). In the development of nurse‐sensitive metrics, this means creating spaces where GPNs can examine their everyday practice, question assumptions and explore together how nursing care contributes within general practice. Appreciative Inquiry supports this relational process by enabling practitioners to reflect together on where nursing care makes a difference and how this contribution might be more clearly articulated. Engaging perspectives from general practitioners, patients and practice administrators further reinforces the relational character of the inquiry by recognising that nursing contribution is understood through its connections with patients, colleagues and the organisational routines of general practice. Through these collaborative processes, emerging understandings are developed through dialogue and reflection (Reason and Bradbury 2008).

Insider Action Research brings the researcher into close relationship with the field of practice under examination. This positioning can support trust and contextual understanding and shared engagement, while also requiring ongoing reflexivity regarding assumptions, power dynamics and professional relationships within the field (Coghlan 2019b; Dwyer and Buckle 2009). Insider positioning is therefore not treated as a source of unexamined authority, but as a position of responsibility that requires ongoing reflexive scrutiny. Consistent with Action Research traditions, reflexive awareness of these dynamics forms part of the inquiry process and informs how dialogue and interpretation evolve over time (Reason and Bradbury 2008). Through these relational processes, a form of inquiry is undertaken in which participation, collaboration and reflexive awareness are central to both the development of knowledge and the demonstration of rigour.

### 7.3 Responsibility for Transparency in the Appreciative Inquiry Process

The quality of the insider action research appreciative inquiry process is demonstrated through transparent, iterative cycles of dialogue, reflection and collective interpretation. Within Responsible Action Research process, rigour is achieved through making visible how understanding develops over time and how emerging interpretations are questioned, refined and translated into potential action. For metrics to remain credible and practice‐grounded, the movement from everyday practice experience to categorisation of quality nursing activities must be explicit.

Ethical engagement is also part of process transparency. In Responsible Action Research, consent is not treated as a single procedural event but as an ongoing relational commitment, requiring continued attention to confidentiality, voluntary participation and participants' right to withdraw (Bergum and Dossetor 2005). This ongoing ethical attentiveness is essential because collaborative inquiry depends on trust, openness and the capacity of participants to question, refine and challenge emerging interpretations safely.

Movement through the inquiry process reflects the broader Action Research cycle in which participants collectively construct understandings of practice, consider potential actions, reflect on their implications and utility in practice over time (Reason and Bradbury 2008). By making visible how experiences are explored, interpreted and translated into candidate domains and indicators, the inquiry demonstrates the iterative and collaborative processes through which knowledge is generated within Responsible Action Research. Insights generated through various forms of data collection can stimulate further reflection and discussion. These feedback loops enable participants to examine emerging interpretations together and refine shared understandings over time. In this way, knowledge develops through collaborative inquiry rather than through analysis conducted independently by the researcher.

Reflexive practice is embedded throughout the inquiry process including ongoing first‐person reflection which enables the researcher to examine how assumptions, interpretations and decisions shape the inquiry. Making these reflexive processes visible addresses critiques that action‐oriented research can appear methodologically opaque, and demonstrates how knowledge emerges through cycles of participation, reflection and action (Bradbury‐Huang 2010).

Moreover, through systematic documentation of meetings, reflections and evolving interpretations, ideas on how to develop and how to categorise quality nursing activities from shared understanding can be demonstrated. In this way, the development of potential nursing quality indicators is grounded in shared interpretation. Such transparency and collaborative engagement reflect the process dimension of rigour where the credibility of action research depends on making visible how knowledge is developed through its cycles of inquiry.

### 7.4 Responsibility for Outcomes in the Appreciative Inquiry: Action, Knowledge and Sustainability

Action Research is concerned not only with improving practice but also with contributing to knowledge beyond the immediate setting (Casey et al. 2018; Reason and Bradbury 2008). Therefore, responsibility for outcomes extends beyond the development and implementation of nurse‐sensitive metrics to include consideration of their longer term implications. By translating aspects of everyday clinical nursing work into practice‐relevant indicators, the co‐developed nurse‐sensitive metrics can make the contribution of general practice nursing more visible within the practice.

Alongside this practical objective, a deeper understanding of how quality nursing care is recognised and enacted within general practice is achieved. Through collaborative reflection on practice experiences, various aspects of nursing contribution, that are not easily captured through conventional measurement systems, can be examined and more clearly articulated. These include both visible activities and less visible dimensions of care, such as relational work, communication, clinical interpretation, continuity, anticipatory action and the patient's experience of nursing care. This process contributes to a clearer conceptual articulation of nursing quality in general practice, providing a foundation from which meaningful indicators may be developed.

Responsible Action Research inquiry offers methodological insight into how insider Action Research, implemented through Appreciative Inquiry, can support collaborative exploration of complex aspects of professional practice within general practice. While the focus is on the development of practice‐informed indicators, at a conceptual level, the inquiry contributes to a clearer understanding of how nursing quality within general practice is recognised, interpreted and enacted in everyday clinical nursing practice. In this way, sustainability is further addressed by recognising that measurement systems can influence practice. Indicators that prioritise easily measurable activities may inadvertently narrow the scope of care or shift attention away from relational aspects of practice. By grounding metric development in collaborative inquiry, this mitigates this risk, thereby ensuring that what is measured reflects what is valued within practice.

## 8 Implications

The implications of this argument for the co‐creation of nursing metrics in general practice extend beyond the production of a new set of indicators. A central implication is that nurse‐sensitive metrics in general practice should not begin by assuming that nursing metrics developed outside this context can adequately represent general practice nursing. Instead, measurement should be developed from within practice, through inquiry into how nursing care is enacted, interpreted and valued in this setting. A Responsible Action Research approach therefore draws attention not only to what is eventually measured, but to the transparency and accountability of the process through which nursing contribution becomes known, named and represented. This reframes metric development as a practice‐development process through which nursing work is collaboratively explored, articulated and responsibly made visible.

Methodologically, the paper suggests that combining Insider Action Research with Appreciative Inquiry offers an engaging and affirmative way of involving practitioners in this process. Rather than positioning practitioners as respondents to externally defined measures, this approach enables them to take ownership of the inquiry, draw on their experiential knowledge and contribute to the development of a shared conceptual language for general practice nursing. For researchers and health system leaders, this challenges assumptions within performance systems that privilege what is readily measurable over what is meaningful, and invites more responsible ways of representing nursing work within healthcare data and quality systems.

## 9 Limitations

The argument developed in this paper is situated within Irish general practice, where nursing roles, policy structures, practice organisation and data systems shape how nursing's contribution is understood and recorded. While the methodological principles may be relevant to other community‐based and primary care nursing contexts, transferability will depend on the extent to which readers recognise similarities with their own professional and organisational settings.

Responsible Action Research also requires time, trust, sustained engagement and organisational support. These conditions may be difficult to secure in busy, resource‐constrained general practice environments. While such investment is central to responsible and relational inquiry, it may limit how easily this approach can be implemented across different practice settings. The insider position of the researcher brings both strengths and challenges. Familiarity with general practice may support trust and contextual understanding, but it may also make taken‐for‐granted aspects of everyday nursing practice harder to question or recognise. Existing professional relationships may influence participation and openness, requiring ongoing reflexive attention to role boundaries, power dynamics, participant voice and transparency in how interpretations are developed.

## 10 Conclusion

This paper argues that efforts to make nursing visible in general practice must begin with inquiry into how nursing practice is enacted, experienced and valued within the settings where care is delivered. Drawing on Appreciative Inquiry within an Insider Responsible Action Research approach, the paper has advanced a methodological argument for developing nurse‐sensitive metrics through shared inquiry into everyday practice. This moves metric development beyond the identification of measurable activities toward consideration of how nursing contribution becomes understood, articulated and responsibly represented.

Knowledge of nursing's contribution is understood here as emerging through iterative cycles of dialogue, reflection and shared interpretation. Responsible Action Research foregrounds the processes through which this knowledge is generated. By engaging with an array of interest‐holders the plurality of knowing is upheld, with multiple perspectives contributing to a more nuanced understanding of nursing practice. This participatory process enables aspects of nursing care that are often tacit to be articulated and considered as potential indicators of quality.

Responsible Action Research is therefore conceptualised not only as a method for generating knowledge but as a site of ethical and relational practice. The processes through which participants engage with one another through dialogue, reflection and shared exploration reflect the relational aspects of nursing care that this paper has argued are central to making nursing visible. In this sense, the inquiry itself can embody a process of care, where participants collectively examine practice, question assumptions and develop new understandings. Through this process, the ethical and relational commitments underpinning Responsible Action Research are not only described but enacted, shaping both the knowledge generated and the conditions under which it is produced. Making these dimensions of rigour explicit is particularly important in a paper concerned with the development of quality nursing care metrics. Without attention to context, relationships, process and outcomes, indicators risk reflecting what is easiest to measure rather than what is most relevant to practice. Responsible Action Research addresses this concern by positioning metric development as a transparent and accountable process of knowledge generation, rather than as a technical exercise of selecting measures.

The methodological contribution of this paper lies in demonstrating how Responsible Insider Action Research, as a practice‐engaged inquiry, can support the development of nurse‐sensitive metrics that remain credible to practitioners while also informing wider health system measurement frameworks. In doing so, the paper contributes methodologically to Action Research scholarship by articulating a responsible framework that integrates Insider Action Research, Appreciative Inquiry and the QuARC. This integration foregrounds context, relational engagement, process transparency and theory development as central to the generation of credible and practice‐grounded knowledge. It also contributes to ongoing debates concerning nurse‐sensitive indicators by demonstrating that what becomes measurable is inseparable from how knowledge of nursing practice is produced, whose perspectives are included and which indicators of care are recognised as meaningful. Although grounded in Irish general practice, the methodological argument has relevance for other community‐based and integrated primary care nursing roles internationally. At its core, this Responsible Action Research approach reframes the purpose of measurement itself. Rather than asking how nursing care can be fitted into existing systems of measurement, it asks how measurement can be developed from within practice to reflect the nature, quality and contribution of nursing care. In doing so, the paper questions dominant positivist approaches to measurement of nursing quality and positions knowledge of nursing practice as relational, context‐dependent and co‐constructed.

What becomes visible in healthcare data systems is never neutral; it reflects decisions about what counts as evidence, whose perspectives matter and which dimensions of care are recognised as quality. Responsible Action Research offers a way of approaching nursing metric development as a collective, reflexive and ethically accountable process. By foregrounding ethical, relational, reflexive and anticipatory dimensions of the inquiry, Responsible Action Research helps ensure that measurement remains connected to what is meaningful within practice. In doing so, the paper reframes metric development in nursing as an epistemic and ethical undertaking, contributing to debates on how nursing work is understood, represented and made visible within healthcare systems. This offers a way of making nursing work more visible without reducing its complexity, while supporting the development of practice‐relevant metrics grounded in the realities of general practice nursing.

## Artificial Intelligence Disclosure Statement

The authors have no use of Artificial Intelligence to disclose.

## Ethics Statement

Ethical approval was not required for this manuscript, as it does not report empirical research involving human participants.

## Conflicts of Interest

The authors declare no conflicts of interest.

## Data Availability

Data sharing is not applicable to this article as no datasets were generated or analysed during the current study.
